# Triglycerides/Glucose and Triglyceride/High-Density Lipoprotein Cholesterol Indices in Normal and Preeclamptic Pregnancies: A Longitudinal Study

**DOI:** 10.1155/2018/8956404

**Published:** 2018-08-06

**Authors:** Natalia Elvira Poveda, María Fernanda Garcés, Aquiles Enrique Darghan, Silvia Alejandra Blanco Jaimes, Estefania Pulido Sánchez, Luz Amparo Díaz-Cruz, Carmen Doris Garzón-Olivares, Mario Orlando Parra-Pineda, Alejandro Antonio Bautista-Charry, Edith Ángel Müller, Héctor Fabio Sandoval Alzate, Luis Miguel Maldonado Acosta, Elizabeth Sanchez, Ariel Iván Ruíz-Parra, Jorge Eduardo Caminos

**Affiliations:** ^1^Department of Physiology, School of Medicine, Universidad Nacional de Colombia, Bogota, Colombia; ^2^Department of Obstetrics and Gynecology, School of Medicine, Universidad Nacional de Colombia, Bogota, Colombia; ^3^Department of Human Nutrition, School of Medicine, Universidad Nacional de Colombia, Bogota, Colombia; ^4^Endocrine Unit-Internal Medicine, School of Medicine, Universidad Nacional de Colombia, Bogota, Colombia

## Abstract

Metabolic changes have been correlated with adverse pregnancy outcomes. The aim of the present study is to determine the TyG and TG/HDL-c indices in a cohort of healthy pregnant (*n* = 142), preeclamptic (*n* = 18), and healthy nonpregnant women (*n* = 56). Preeclamptic women were selected from the same cohort. Pregnant women were followed during three periods of pregnancy and postpartum. The results showed a significant increase in the values of TyG and TG/HDL-c (*p* < 0.01) as pregnancy progresses, without significant differences between healthy and preeclamptic women. TyG and TG/HDL-c indices are significantly low in nonpregnant and three months' postpartum women when compared with each gestational period studied. TyG and TG/HDL-c indices are positively correlated with HOMA-IR in the early and middle pregnancy (*p* < 0.05). Multiple linear regression using the TyG and TG/HDL-c indices as dependent variables showed that TyG index was significantly associated with HOMA-IR, gestational age, HDL-c, TC, LDL, fasting insulin, and mean BP (*p* < 0.001); meanwhile, TG/HDL-c index was only associated with HOMA-IR (*p* < 0.0242) and gestational age (*p* < 0.001). In conclusion, the TyG and TG/HDL-c indices could be useful in monitoring insulin resistance during pregnancy.

## 1. Introduction

Pregnancy is a state of multiple and critical changes in the morphology and physiology of women, which play a fundamental role in meeting the mother's basal needs and the requirements of the developing fetus [1]. Among the maternal physiological adaptations are the increase of body fat in order to increase the energy reserve, the transient decrease in insulin sensitivity by 40–50% towards the second and third trimesters, and thus the increase in circulating lipid and amino acid concentrations. Additionally, there is an increase in triglyceride (TG) levels mediated by inactivation of hepatic lipase secondary to the action of high-density lipoprotein (HDL) and to the elevation of very low-density lipoprotein (VLDL). Together, these changes are due to the increase in estrogen levels during pregnancy [2, 3]. The elevated estrogen levels during gestation result in an increased hepatic synthesis of triglyceride-rich VLDL and reduction of the removal of lipoprotein triglycerides due to low activities of the adipose lipoprotein lipase and hepatic lipase (HL) [4, 5].

In nonpregnant patients with a body mass index (BMI) in the range of overweight or obesity, insulin resistance is a predisposing factor to generate metabolic syndrome and related diseases such as dyslipidemia, hypertension, and diabetes [6]. In pregnant women, there are several proposed mechanisms that lead to a state of insulin resistance. Different proinflammatory adipocytokines, produced by the placenta and in the adipose tissue, lead to the development of low-grade chronic inflammation state [7]. The progressive accumulation of adipose tissue has a strong correlation with the increase of leptin levels during pregnancy. This could be considered as a “leptin resistance” state [8, 9], whose objective in the normal state is to improve the availability of glucose for the fetus and to offer alternative sources of energy for the pregnant woman.

Hypertriglyceridemia and hypercholesterolemia are physiological conditions in the second trimester of gestation [10]. Two groups, Khouly et al. and Wang et al. showed that during the first trimester of gestation, elevated levels of total cholesterol (TC), TG, and LDL-c and low serum HDL-c levels are correlated with adverse pregnancy outcomes such as preeclampsia [6, 11]. This pathology is related to the alteration in the process of modification of spiral arterioles and the synthesis of proinflammatory and antiangiogenic factors derived from the placenta, which lead to an endothelial dysfunction similarly as a hyperlipidemic state [12].

Identification of preconceptional cardiovascular risk is limited but it is important, using methods that are easily applicable and accessible for physicians, cost-effective, and with adequate diagnostic performance [6, 8]. In the literature, there is currently a broad description of indices used for this purpose although most of them have been developed in the adult population without being validated in pregnancy [6, 13]. Thus, the aim of the present study is to determine the triglyceride/glucose ratio (TyG) and triglyceride/high-density lipoprotein cholesterol (TG/HDL-c) indices in a cohort of women with normal gestation and in a group of pregnant women who developed preeclampsia and to correlate these indices with HOMA-IR, biochemical, anthropometric, and hormonal variables. Therefore, this study may contribute to identify key factors involved in adverse pregnancy outcomes due to metabolic complications in a more cost-effective manner and to take early preventive actions during the preconception and gestational periods.

## 2. Subjects and Methods

### 2.1. Ethics Statement

The present study was approved by the Ethics Committee of the School of Medicine of the Universidad Nacional de Colombia, in accordance with the ethical guidelines established by the Declaration of Helsinki. All study participants accepted their voluntary participation through the signing of an informed consent. The women included in the study were attended by health care personnel from the Department of Obstetrics and Gynecology of the School of Medicine of the Universidad Nacional de Colombia, in Engativá Hospital in the city of Bogotá, D.C.

### 2.2. Subjects and Study Design

The present is a prospective cohort study. It included a group of healthy pregnant women (*n* = 142) followed during three periods of pregnancy, early (12.2, range: 10.5–14.4 weeks of gestation), middle (24.3, range: 23.3–27.3 weeks of gestation), and late (34.5, range: 33.3–38.6 weeks of gestation) and at three months postpartum who attended visits between May 2012 and November 2015. In addition, a group of healthy nonpregnant women (*n* = 56) was included in the study.

The selection of the healthy pregnant group was carried out according to the recommendations of the International Federation of Clinical Chemistry (IFCC) [14], following two selection phases: a priori and a posteriori. In the first phase, healthy women with gestational age between weeks 10 and 12.6 determined by ultrasonography were selected, parity from 0 to 4, single fetus, with a BMI between 17 and 29.9 kg/m^2^, who reported no previous history of chronic diseases, not currently taking medication, and nonsmokers and did not consume alcohol habitually. In the second phase of selection, which was after the maternal-perinatal outcome, only normal-course pregnancies were included, women who delivered at term, babies with normal weight at birth who did not present abnormalities or fetal malformations, and women who did not develop pathologies associated with pregnancy.

Additionally, 18 pregnant women who developed nonsevere preeclampsia belonging to the same cohort were selected. The diagnosis of preeclampsia was made in accordance with the recommendations of the American College of Obstetricians and Gynecologists [15]. Finally, the present study included 56 healthy nonpregnant women with regular ovulatory menstrual cycles (progesterone > 3.0 ng/ml), normal BMI (between 18.5 and 24.9 kg/m^2^), not currently breastfeeding, nonsmokers, without previous history of psychoactive substance or habitual alcohol use, and not using medications that alter glucose tolerance (*β*-adrenergic agonists, *β*-blockers, corticosteroids, or other drugs that can affect the metabolism).

### 2.3. Laboratory Assays

In each period of pregnancy and in nonpregnant women, blood was taken after 10–12 hours of fasting, between 7:00 and 8:00 am. Nonpregnant women were studied during the two phases of the menstrual cycle, follicular phase (3rd to 5th day of cycle) and luteal phase (20th to 22nd day of cycle). Additionally, in nonpregnant women, at each phase of the menstrual cycle, two blood samples were taken between 9 am and 12 m, with a half-hour interval, in order to report the average progesterone concentration [16]. BD Vacutainer dry tubes (5 ml) were used to draw blood. The blood samples were left at room temperature for 20 minutes, and the coagulated blood was centrifuged at 1000*g* for 10 minutes at 4°C. Serum was stored at −80°C until analysis.

Basal glucose, total cholesterol, HDL cholesterol, LDL cholesterol, and triglycerides were determined (Spinreact, Santa Coloma, Spain). VLDL cholesterol was calculated as one-fifth of triglycerides [17]. Basal insulin was determined by chemiluminescence assay (Roche Elecsys 1010 Immunoanalyzer Boulder, Colorado, United States), and the ultrasensitive C-reactive protein was determined by immunoturbidimetry BS-400 Chemistry Analyzer (Mindray, Shenzhen, China). The HOMA-IR (homeostasis model assessment) index described by Matthews et al. was calculated with the values of basal glucose and insulin concentration [18]. The values of the TyG and TG/HDL-c indices were calculated as previously described [19]. The QUICKI (quantitative insulin sensitivity check index) described by Katz et al. was calculated using the formula proposed by them QUICKI = 1/[log(I_0_) + log(G_0_)], including the fasting plasma glucose and insulin levels of our groups of study [20]. Serum progesterone levels were determined by immunoassay (Roche Elecsys 1010 Immunoanalyzer Boulder, Colorado, United States).

### 2.4. Statistical Analysis

The statistical tests were carried out with the statistical program R (version 3.1.1). Data with normal distribution are described as mean ± SD (standard deviation), while data with a nonnormal distribution are presented as median and interquartile range. The statistical differences between paired samples and the comparison between pregnant women in the different periods of pregnancy and the postpartum period, as well as the differences between the follicular and luteal phases, were evaluated through the Wilcoxon signed-rank test. The differences between the medians of healthy pregnant women and pregnant women with preeclampsia were evaluated through the Mann–Whitney test (Mann–Whitney *U* test) directed to independent samples.

The Spearman correlation coefficient was determined between the TyG, TG/HDL-c, and HOMA-IR indices and the serum levels of the biochemical and anthropometric variables in each of the gestation periods. Multiple correlations were determined, throughout the three periods of gestation, using the TyG, TG/HDL-c, and HOMA-IR indices as dependent variables and the demographic variables, clinical, and biochemical characteristics of the study population as independent. Values with statistical significance are presented as ^∗^ *p* < 0.05, ^∗∗^ *p* < 0.01, and ^∗∗∗^ *p* < 0.001.

## 3. Results

**Figure 1:**
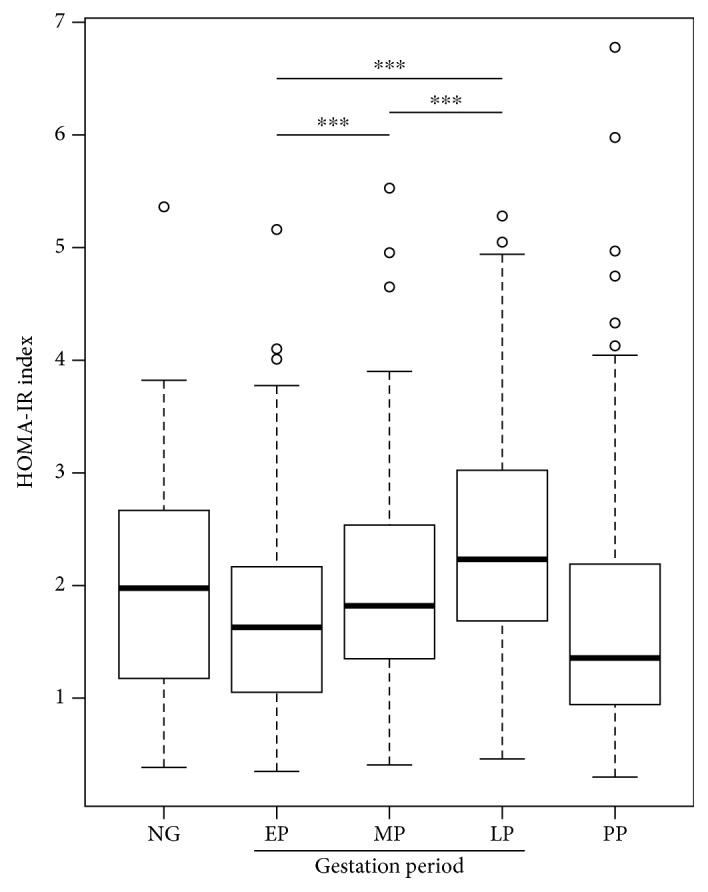
HOMA-IR index during pregnancy. Box-and-whisker plot with median value, interquartile range, and lower and upper values for each group of subjects, healthy women during three stages of pregnancy and three months postpartum and a group of healthy nonpregnant women. ^∗∗∗^Statistically significant difference between the groups.

**Figure 2:**
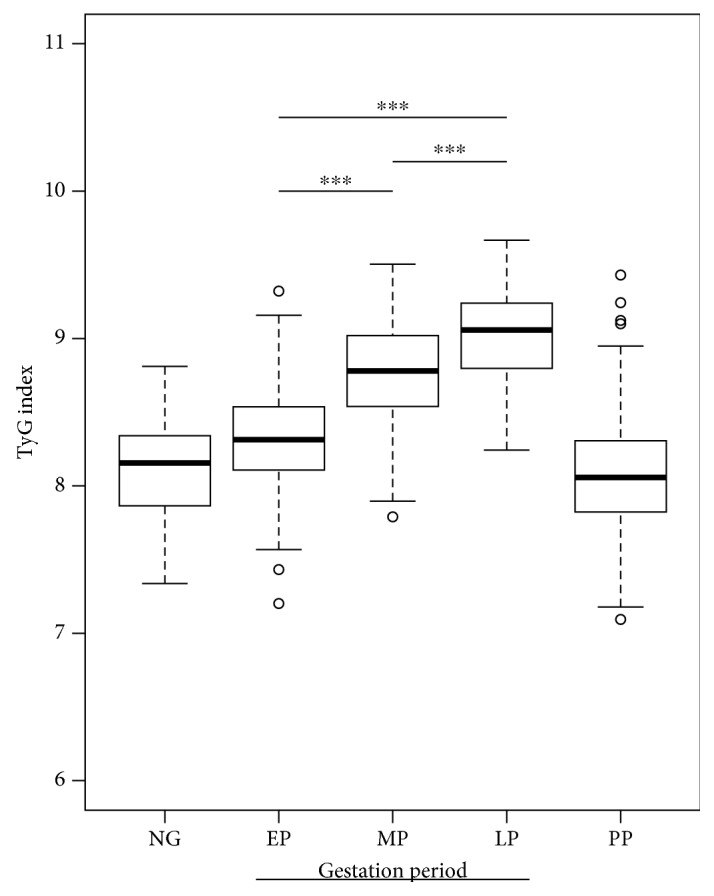
TyG index during pregnancy. Box-and-whisker plot with median value, interquartile range, and lower and upper values for each group of subjects, healthy women during three stages of pregnancy and three months postpartum and a group of healthy nonpregnant women. ^∗∗∗^Statistically significant difference between the groups.

**Figure 3:**
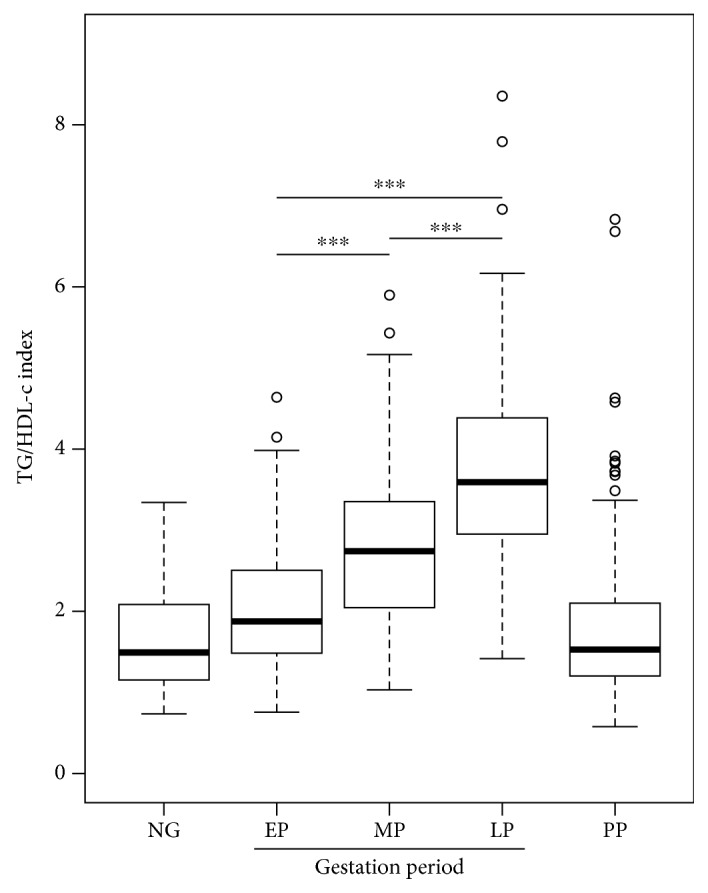
TG/HDL-cc index during pregnancy. Box-and-whisker plot with median value, interquartile range, and lower and upper values for each group of subjects, healthy women during three stages of pregnancy and three months postpartum and a group of healthy nonpregnant women. ^∗∗∗^Statistically significant difference between the groups.

Firstly, the demographic, clinical, and biochemical characteristics of women, healthy pregnant women (Table 1), women who developed mild preeclampsia (Supplementary Table 1), and nonpregnant women (Supplementary Table 2) can be observed. As previously described, significant changes were observed throughout gestation, among others, in BMI (*p* < 0.01), fasting glucose (*p* < 0.01), triglycerides (*p* < 0.01), total cholesterol (*p* < 0.01), LDL-cholesterol (*p* < 0.01), fasting insulin (*p* < 0.01), HOMA-IR (*p* < 0.01), and QUICKI (*p* < 0.01) (Table 1 and Figure 1). Additionally, in the present study, a significant increase in the values of TyG (*p* < 0.01) (Figure 2) and TG/HDL-c (*p* < 0.01) (Figure 3) throughout the three periods of gestation in both normal and preeclamptic women was described for the first time (Table 1 and Supplementary Table 1).

Significant differences were observed over the three periods studied between normal and preeclamptic pregnant women in relation to BMI (*p* < 0.01), systolic BP (*p* < 0.01), and diastolic BP (*p* < 0.01) (Supplementary Table 3). Additionally, a significant difference was observed between the two groups in the second gestation period in the levels of C-reactive protein (*p* < 0.01), fasting insulin (*p* < 0.01), HOMA-IR (*p* < 0.01), and QUICKI (*p* < 0.01) (Supplementary Table 3).

On the other hand, in Supplementary Table 4, significant differences were observed between normal nonpregnant women and pregnant women in each of the three periods of gestation studied: differences in BMI (*p* < 0.01), systolic BP (*p* < 0.01), diastolic BP (*p* < 0.01), mean BP (*p* < 0.01), fasting glucose (*p* < 0.01), triglycerides (*p* < 0.01), total cholesterol (*p* < 0.01), HDL-cholesterol (*p* < 0.01), fasting insulin (*p* < 0.01), HOMA-IR (*p* < 0.01), TyG index (*p* < 0.01), and TG/HDL-c index (*p* < 0.01).

As it is shown in Table 2, TyG and TG/HDL-c indices positively and significantly correlated with HOMA-IR in the first (*r* = 0.274, *p* < 0.05) (*r* = 0.168, *p* < 0.05) and second (*r* = 0.316, *p* < 0.05) (*r* = 0.258, *p* < 0.05) periods of pregnancy, respectively. On the other hand, there were no significant correlations between TyG and TG/HDL-c with HOMA-IR in the third period of gestation (Table 2). Additionally, TyG index correlated positively and significantly with total cholesterol (*r* = 0.491, *p* < 0.05) (*r* = 0.345, *p* < 0.05) (*r* = 0.413, *p* < 0.05) in the three periods of pregnancy studied (Table 2).

Multiple linear regression was performed using the TyG index as a dependent variable and HOMA-IR, BMI, gestational age, HDL-cholesterol, total cholesterol, LDL-cholesterol, fasting insulin, and mean BP as independent variables. The results showed that TyG index was associated with HOMA-IR (*p* < 0.001), gestational age (*p* < 0.001), HDL-cholesterol (*p* < 0.001), total cholesterol (*p* < 0.001), LDL-cholesterol (*p* < 0.0001), fasting insulin (*p* < 0.001), and mean BP (*p* < 0.001) but not with BMI (Supplementary Table 5).

Also, multiple linear regression was realized to TG/HDL-c index as a dependent variable and LDL-cholesterol, total cholesterol, gestational age, HOMA-IR, BMI, fasting insulin, and mean BP as independent variables. In this case, the results showed that TG/HDL-c index was only associated with HOMA-IR (*p* < 0.0242) and gestational age (*p* < 0.001) but not with LDL-cholesterol, total cholesterol, BMI, fasting insulin, and mean BP (Supplementary Table 5). Finally, HOMA-IR index as a dependent variable, was associated with BMI (*p* < 0.001) and triglycerides (*p* < 0.0214), but not with gestational age, HDL-cholesterol, total cholesterol, LDL-cholesterol, fasting insulin, mean BP, or BMI (Supplementary Table 5).

## 4. Discussion

In the present study, the values of TyG and TG/HDL-c are reported for the first time during three periods of normal gestation and in preeclamptic women. It is observed that the value of both indices increases significantly with the advance of gestation, both in normal and preeclamptic pregnant women. On the other hand, the value of the TyG and TG/HDL-c indices is significantly low, both in healthy nonpregnant women and in women three months after delivery. There were no significant differences in the value of the indices between normal pregnancy and preeclampsia in the three periods of gestation. In addition, the TyG and TG/HDL-c indices showed a strong positive association with the HOMA-IR index, in the first two periods of gestation. In the multivariate analysis, throughout gestation, the TyG and TG/HDL-c indices, as dependent variables, correlate significantly with the HOMA-IR and with the gestational age as independent variables.

Previous studies have shown that during pregnancy, serum levels of triglycerides, total cholesterol, LDL-c, and VLDL-c increase [21–23]. In contrast, HDL-c levels rise between the first and second trimesters of pregnancy but decrease in the third trimester [21, 22]. In the present study, a behavior similar to that previously described was observed regarding the lipid profile changes during normal gestation, changes that at the same time lead to variations of the values of TyG and TG/HDL-c indices along the gestation.

Different studies have shown that during normal pregnancy, there is a significant reduction in insulin sensitivity, being lower in the third trimester compared to the first and second trimesters of pregnancy and when compared with healthy nonpregnant women [24–27]. Catalano et al. described an increase in basal insulin levels in about 65% by the third trimester of pregnancy [27], while Sonagra et al. reported an increase in basal insulin levels in pregnant women of 61% in 3rd trimester and 29% in 2nd trimester when compared with nonpregnant controls [25]. The results of the present study showed the same behavior in the basal insulin levels described in the previous studies as well as the metabolic, biochemical, physiological, and hormonal changes throughout the gestation. These changes are associated with the increase in circulating levels of human placental lactogen (hPL), estrogen, progesterone, cortisol, human placental growth hormone (hPGH), tumor necrosis factor *α* (TNF*α*), and different interleukins [7].

In the present study, it was observed that during pregnancy, the values of the QUICKI decreased significantly during pregnancy, while the values of the HOMA-IR index increased, as previously described in normal gestation [25]. Furthermore, in the present study, it was shown that both the QUICKI and HOMA-IR indices have a significant correlation with the TyG and TG/HDL-c indices during the first two trimesters of pregnancy. Finally, the multiple correlation analysis showed that the HOMA-IR index is significantly correlated with the TyG and TG/HDL-c indices during the first and the second trimester, but not in the third trimester.

Cohen et al. and Kirwan et al. determined the correlation between hepatic insulin resistance (HOMA-IR) and the hyperinsulinemic euglycemic clamp during pregnancy [28, 29]. The results of these studies showed that there is a significant correlation between the HOMA-derived S% and the clamp-derived GRD [28, 29]. It is important to consider that Cohen et al. and Kirwan et al. stated that the clamp technique examines the individual response to the state of hyperinsulinemia, while the HOMA reflects the steady state reached in the fast. In addition, Cohen et al. and Kirwan et al. consider that HOMA is more related to the determination of hepatic insulin sensitivity and not to peripheral tissues such as the skeletal muscle, a particular situation that occurs at the end of pregnancy. On the other hand, Katz et al. describes that HOMA and clamp lose linearity with the increase in insulin resistance and it is not recommended in patients with advanced diabetes [20]. Late gestation is a condition of high insulin resistance, and the same phenomenon described by Katz et al. [20] could occur. In this way, the absence of statistical significance of the correlation between the HOMA-IR and TyG and HOMA-IR and TG/HDL-c indices, found in the third gestational period in the present study, may be due to the greater insulin resistance that occurs in the muscle and not in the liver at this stage of pregnancy. In conclusion, it is necessary to determine and validate, at the end of pregnancy, the correlation between the TyG and TG/HDL-c indices against the gold standard for determining insulin sensitivity, the hyperinsulinemic euglycemic clamp to confirm this hypothesis.

Different studies have proposed the TyG, TG/HDL-c, and HOMA-IR indices as cost-effective surrogate markers to estimate IR among adults in comparison with the euglycemic hyperinsulinemic clamp [30, 31]. In addition, it has been shown that the TyG, TG/HDL-c, and HOMA-IR indices reflect the metabolic condition of the individual and predict the development of diabetes [32–36]. Recently, von Bibra et al. [37], in a cohort of European patients, proposed the cutoff point for the TG/HDL-c ratio to identify individuals at risk for insulin resistance and metabolic syndrome, at TG/HDL-c ratio > 2.8 in men and >1.9 in women. McLaughlin et al., in a study developed in the Mexican and white American population, proposed a TG/HDL-c ratio ≥ 3.5 to identify insulin-resistant patients with increased risk of cardiovascular disease [30]. Additionally, Li et al., in a study developed in the population of the United States, found a cutoff for the TG/HDL-c ratio of 3.0 for non-Hispanic whites and Mexican Americans and 2.0 for non-Hispanic blacks, to predict the presence of insulin resistance and dyslipidemia [38].

In this way, it is clear that the cutoff points to predict insulin resistance, based on the TG/HDL-c ratio, are associated with racial/ethnic component and should be studied in different populations. On the other hand, in the present study, it is observed that during pregnancy, the values of the TG/HDL-c index during pregnancy, even exceed the cutoff values of the TG/HDL-c ratio from the first trimester. The values that have been reported as predictors of the increased risk of clinical syndromes related to defect in insulin action and cardiovascular disease. In this way, it is important to determine the cutoff points per quarter for the different populations, in order to predict risks associated with metabolic diseases from the early stages of pregnancy.

Lee et al. determined that during early pregnancy (gestational age of less than 14 weeks), the values of TG/HDL-c and LDL-c/HDL-c ratios are higher in women at risk of developing gestational diabetes mellitus [GDM] when compared with women of normal gestation [34]. Additionally, dos Santos-Weiss et al. [39] showed that the logarithm of the ratio TG/HDL-c could be used to identify pregnant women with low risk of gestational diabetes before 24 weeks of gestation. In the present study, the triglycerides/HDL-cholesterol ratio does not discriminate between healthy pregnant women and pregnant women who developed mild preeclampsia, in any of the periods of gestation studied.

It has been shown that the TyG index is a surrogate marker of the degree of insulin resistance and predicts the risk of developing diabetes in men and women, both in obese and nonobese subjects [34]. Unger et al. determined as a good discriminant of metabolic syndrome, a cutoff point for the TyG index of 8.8 in men and 8.7 in women [13]. Recently, Lee et al. estimated in individuals metabolically obese but with normal weight a cutoff point for the TyG index above 8.82 for men and 8.73 for women [32]. In this way, in the present study, it can be observed that from the second gestation period, the TyG index value in healthy pregnant women reaches values that, when compared with previous studies in nonpregnant women, are above the values of women with metabolic syndrome. The values of cutoff throughout the gestation allow the determination of possible values of normality in the TyG index throughout the gestation, but they must be estimated for each race/ethnic group. The determination of the cutoff points throughout the gestation of the TyG and TG/HDL-c indices, could serve for the diagnosis and monitoring of the pregnant woman's metabolic condition.

## 5. Conclusions

In the present study, the values of TyG and TG/HDL-c were determined in a prospective cohort study during three periods of gestation, which once again confirm the concept of Mouzon and Lassance [1], who state that normal pregnancy is characterized as a “diabetogenic state.” Additional studies could contribute in a useful and economical way to the metabolic and insulin resistance monitoring of the pregnant woman, using the TyG andTG/HDL-c indices. In this way, it is recommended the development of complementary studies to establish cutoff points of TyG and TG/HDL-c per each trimester in normal pregnant women, overweight pregnant women, and low weight according to the racial/ethnic groups and discriminated by age.

## Figures and Tables

**Table 1:** Demographic, clinical, and biochemical characteristics of healthy women during pregnancy and three months postpartum.

Variables	Healthy women (*n* = 142)				
	EP	MP	LP	PP	ANOVA test^∗^
	Mean ± SD	Mean ± SD	Mean ± SD	Mean ± SD	*p*-value
Age (years)	25.86 ± 5.91	NA	NA	NA	NA
Gestational age at blood sampling (weeks)	12.25 ± 0.77	24.55 ± 0.67	34.73 ± 0.85	NA	*p* < 0.01
Height (meters)	1.58 ± 0.05	NA	NA	NA	NA
BMI (kg/m^2^)	22.7 ± 2.8	24.6 ± 2.7	26.4 ± 2.8	23.69 ± 2.91	*p* < 0.01
Systolic blood pressure (mmHg)	97.7 ± 9.7	97.0 ± 10.6	99.3 ± 9.3	103.22 ± 13.5	*p* > 0.05
Diastolic blood pressure (mmHg)	62.6 ± 6.6	61.4 ± 6.4	62.4 ± 7.3	66.08 ± 9.24	*p* > 0.05
Mean blood pressure (mmHg)	74.3 ± 6.8	73.3 ± 7.0	74.7 ± 7.2	78.67 ± 8.84	*p* > 0.05
Fasting glucose (mg/dl)	77.9 ± 8.1	74.1 ± 6.4	73.5 ± 6.8	80.76 ± 7.43	*p* < 0.01
Triglycerides (mg/dl)	112.0 ± 39.6	181.0 ± 58.1	236.2 ± 68.9	87.44 ± 40.59	*p* < 0.01
Total cholesterol (mg/dl)	165.5 ± 33.1	222.1 ± 41.4	244.2 ± 49.6	170.35 ± 29.9	*p* < 0.01
HDL-cholesterol (mg/dl)	56.0 ± 10.4	66.7 ± 12.3	64.8 ± 12.8	51.12 ± 9.62	*p* < 0.01
LDL-cholesterol (mg/dl)	102.8 ± 31.0	138.1 ± 42.4	155.4 ± 48.9	111.98 ± 32.5	*p* < 0.01
C-reactive protein (mg/l)	5.0 ± 2.9	4.8 ± 2.8	5.6 ± 3.4	3.45 ± 3.40	*p* > 0.05
Fasting insulin (*μ*UI/ml)	8.7 ± 4.0	10.7 ± 4.7	12.9 ± 4.9	8.56 ± 5.15	*p* < 0.01
HOMA-IR	1.69 ± 0.8	2.0 ± 0.9	2.4 ± 1.0	1.73 ± 1.13	*p* < 0.01
QUICKI	0.362 ± 0.03	0.352 ± 0.03	0.342 ± 0.02	0.37 ± 0.04	*p* < 0.01
TyG index	8.3 ± 0.37	8.8 ± 0.4	9.0 ± 0.3	8.08 ± 0.43	*p* < 0.01
TG/HDL-c index	2.1 ± 0.8	2.8 ± 1.0	3.8 ± 1.2	1.80 ± 1.01	*p* < 0.01

^∗^Nonparametric ANOVA test. EP: early pregnancy; MP: middle pregnancy; LP: late pregnancy; PP: three months postpartum; BMI: body mass index; HOMA-IR: homeostasis model assessment-estimated insulin resistance; QUICKI: quantitative insulin sensitivity check index; TyG index: triglycerides/glucose; TG/HDL-c index: triglycerides/high-density lipoprotein cholesterol. A *p*-value < 0.05 was considered statistically significant.

**Table 2:** Spearman's correlation coefficients by each trimester of healthy pregnancy.

Variables	Early pregnancy						Middle pregnancy						Late pregnancy					
	HOMA-IR		TyG		TG/HDL-c		HOMA-IR		TyG		TG/HDL-c		HOMA-IR		TyG		TG/HDL-c	
	*r*	*p*-value	*r*	*p*-value	*r*	*p*-value	*r*	*p*-value	*r*	*p*-value	*r*	*p*-value	*r*	*p*-value	*r*	*p*-value	*r*	*p*-value
BMI	0.304	<0.05	0.031	>0.05	0.172	<0.05	0.346	>0.05	0.102	>0.05	0.092	>0.05	0.438	<0.05	0.023	>0.05	0.061	>0.05
Systolic BP	−0.111	>0.05	−0.136	>0.05	−0.050	>0.05	−0.034	>0.05	−0.133	>0.05	−0.066	>0.05	−0.032	>0.05	−0.076	>0.05	0.053	>0.05
Diastolic BP	−0.064	>0.05	0.043	>0.05	0.014	>0.05	0.045	>0.05	−0.119	>0.05	−0.007	>0.05	−0.032	>0.05	−0.119	>0.05	−0.146	>0.05
Mean BP	0.095	>0.05	0.093	>0.05	0.015	>0.05	−0.046	>0.05	−0.141	>0.05	−0.038	>0.05	−0.036	>0.05	−0.113	>0.05	−0.075	>0.05
Fasting glucose																		
Triglycerides	0.148	>0.05					0.211	<0.05					−0.062	>0.05				
Total cholesterol	0.014	>0.05	0.491	<0.05	0.139	>0.05	−0.119	>0.05	0.345	<0.05	0.041	>0.05	−0.010	>0.05	0.413	<0.05	0.056	>0.05
HDL-cholesterol	−0.024	>0.05	0.094	>0.05			−0.070	>0.05	0.049	>0.05			−0.051	>0.05	0.187	<0.05		
LDL-cholesterol	0.085	>0.05	0.276	<0.05	0.061	>0.05	−0.113	>0.05	0.097	>0.05	−0.057	>0.05	0.043	>0.05	0.173	<0.05	0.053	>0.05
C-reactive protein	0.085	>0.05	0.157	>0.05	0.189	<0.05	0.153	>0.05	0.149	>0.05	0.164	>0.05	0.041	>0.05	0.085	>0.05	0.178	<0.05
Fasting insulin			0.227	<0.05	0.173	<0.05			0.263	<0.05	0.236	<0.05			0.034	>0.05	0.055	>0.05
HOMA-IR			0.274	<0.05	0.168	<0.05			0.316	<0.05	0.258	<0.05			0.087	>0.05	0.036	>0.05
QUICKI			−0.339	<0.05	−0.240	<0.05			−0.316	<0.05	−0.232	<0.05			−0.128	>0.05	0.032	>0.05
TyG index	0.274	<0.05					0.316	<0.05					0.087	>0.05				
TG/HDL-c index	0.168	<0.05					0.258	<0.05					0.036	>0.05				

BMI: body mass index; BP: blood pressure; HOMA-IR: homeostasis model assessment-estimated insulin resistance; QUICKI: quantitative insulin sensitivity check index; TyG index: triglycerides/glucose; TG/HDL-c index: triglycerides/high-density lipoprotein cholesterol. A *p*-value < 0.05 was considered statistically significant.

## Data Availability

The data used to support the findings of this study are available from the corresponding author upon request.

## Supplementary Materials

Supplementary Materials The supplementary word file contains five supplementary tables described in the text that support our results. Table 1: demographic, clinical, and biochemical characteristics of preeclamptic woman during pregnancy and three months postpartum. Table 2: demographic, clinical, and biochemical characteristics of healthy nonpregnant women. Table 3: post hoc analyses between healthy pregnant women and preeclamptic women. Table 4: post hoc analyses between healthy pregnant women and healthy nonpregnant women. Table 5: multiple linear regression analysis.
