# Supplementary material for: Triglycerides/Glucose and Triglyceride/High-Density Lipoprotein Cholesterol Indices in Normal and Preeclamptic Pregnancies: A Longitudinal Study
Source: Int J Endocrinol. 2018 Aug 6;2018:8956404. doi: 10.1155/2018/8956404 (PMC6109518; doi:10.1155/2018/8956404)
Supplement: Supplementary Materials — The supplementary word file contains five supplementary tables described in the text that support our results. Table 1: demographic, clinical, and biochemical characteristics of preeclamptic woman during pregnancy and three months postpartum. Table 2: demographic, clinical, and biochemical characteristics of healthy nonpregnant women. Table 3: post hoc analyses between healthy pregnant women and preeclamptic women. Table 4: post hoc analyses between healthy pregnant women and healthy nonpregnant women. Table 5: multiple linear regression analysis. [file 8956404.f1.docx]

**Supplementary table 1.Demographic, Clinical and Biochemical characteristics of preeclamptic woman during pregnancy and three months postpartum**

| **Variables** | **Preeclamptic women (n=18)** | | | | |
| --- | --- | --- | --- | --- | --- |
|  | **EP** | **MP** | **LP** | **PP** | **ANOVA test*** |
|  | **Mean ± SD** | **Mean ± SD** | **Mean ± SD** | **Mean ± SD** | **p-value** |
| Age (years) | 23.17 ± 6.46 | NA | NA | NA | NA |
| Gestational age at blood sampling (weeks) | 12.14 ± 0.69 | 24.39 ± 0.55 | 35.04 ± 0.88 | NA | p<0.01 |
| Height (meters) | 1.58 ± 0.06 | NA | NA | NA | NA |
| BMI (kg/m**^2^)** | 24.2 ± 2.9 | 26.6 ± 2.8 | 29.6 ± 2.7 | 26.65 ± 3.67 | p<0.01 |
| Systolic Blood Pressure (mmHg) | 105.0 ± 7.1 | 104.0 ± 9.1 | 111.1 ± 17.9 | 107.78 ± 13.7 | p>0.05 |
| Diastolic Blood Pressure (mmHg) | 66.9 ± 6.8 | 65.1 ± 7.2 | 65.2 ± 7.8 | 69.28 ± 9.33 | p>0.05 |
| Mean Blood Pressure (mmHg) | 79.6 ± 6.3 | 78.1 ± 6.7 | 80.5 ± 10.3 | 82.11 ± 9.82 | p>0.05 |
| Fasting glucose (mg/dL) | 80.9 ± 6.8 | 77.2 ± 7.5 | 74.6 ± 9.6 | 84.61 ± 7.69 | p<0.05 |
| Triglycerides (mg/dL) | 113.8 ± 49.9 | 173.9 ± 70.6 | 251.9 ± 79.7 | 125.22 ± 79.7 | p<0.01 |
| Total cholesterol (mg/dL) | 172.2 ± 34.5 | 219.7 ± 46.6 | 234.6 ± 51.0 | 186.35 ± 38.6 | p<0.01 |
| HDL-cholesterol (mg/dL) | 56.0 ± 10.4 | 66.7 ± 12.3 | 59.4 ± 17.3 | 47.81 ± 11.31 | p<0.01 |
| LDL-cholesterol (mg/dL) | 123.2 ± 41.1 | 152.8 ± 61.2 | 161.4 ± 66.4 | 138.99 ± 32.6 | p<0.05 |
| C Reactive Protein (mg/L) | 6.2 ± 4.2 | 7.4 ± 3.0 | 7.2 ± 3.6 | 4.59 ± 3.18 | p>0.05 |
| Fasting insulin (µUI/mL) | 17.9 ± 5.9 | 15.4 ± 4.5 | 15.2 ± 6.4 | 14.17 ± 7.53 | p>0.05 |
| HOMA-IR | 3.9 ± 1.5 | 2.9 ± 0.8 | 2.8 ± 1.3 | 2.99 ± 1.74 | p>0.05 |
| QUICKI | 0.334 ± 0.03 | 0.328 ± 0.01 | 0.335 ± 0.03 | 0.33 ± 0.03 | p>0.05 |
| TyG index | 8.3 ± 0.4 | 8.7 ± 0.5 | 9.1 ± 0.5 | 8.40 ± 0.64 | p<0.01 |
| TG/HDL-c index | 2.2 ± 1.0 | 2.9 ± 1.4 | 4.8 ± 2.9 | 2.84 ± 1.84 | p<0.01 |

*Non-Parametric ANOVA test. EP = early pregnancy. MP = middle pregnancy. LP = late pregnancy. PP = three months postpartum. BMI (body mass index); HOMA-IR (homeostasis model assessment-estimated insulin resistance); QUICKI (quantitative insulin sensitivity check index); TyG index (triglycerides/glucose); TG/HDL-c index (triglycerides/high-density lipoprotein cholesterol). A p-value < 0.05 was considered statistically significant.

**Supplementary table 2.Demographic. Clinical and Biochemical characteristics of healthy non-pregnant women**

| **Variables** | **Healthy non-pregnant women (n=56)** |
| --- | --- |
|  | **Mean ± SD** |
| Age (years) | 23.17 ± 6.46 |
| Gestational age at blood sampling (weeks) | 12.14 ± 0.69 |
| Height (meters) | 1.58 ± 0.06 |
| BMI (kg/m**^2^)** | 24.2 ± 2.9 |
| Systolic Blood Pressure (mmHg) | 105.0 ± 7.1 |
| Diastolic Blood Pressure (mmHg) | 66.9 ± 6.8 |
| Mean Blood Pressure (mmHg) | 79.6 ± 6.3 |
| Fasting glucose (mg/dL) | 80.9 ± 6.8 |
| Triglycerides (mg/dL) | 113.8 ± 49.9 |
| Total cholesterol (mg/dL) | 172.2 ± 34.5 |
| HDL-cholesterol (mg/dL) | 56.0 ± 10.4 |
| LDL-cholesterol (mg/dL) | 123.2 ± 41.1 |
| C Reactive Protein (mg/L) | 6.2 ± 4.2 |
| Fasting insulin (µUI/mL) | 17.9 ± 5.9 |
| HOMA-IR | 3.9 ± 1.5 |
| QUICKI | 0.334 ± 0.03 |
| TyG index | 8.3 ± 0.4 |
| TG/HDL-c index | 2.2 ± 1.0 |

EP = early pregnancy. MP = middle pregnancy. LP = late pregnancy. PP = three months postpartum. BMI (body mass index); HOMA-IR (homeostasis model assessment-estimated insulin resistance); QUICKI (quantitative insulin sensitivity check index); TyG index (triglycerides/glucose); TG/HDL-c index (triglycerides/high-density lipoprotein cholesterol).

**Supplementary table 3. Post-hoc analyses between healthy pregnant women and preeclamptic women**

| **Variables** | **EP vs EPPE** | **MP vs MPPE** | **LP vs LPPE** | **PP vs PPPE** |
| --- | --- | --- | --- | --- |
| Age (years) | > 0.05 | NA | NA | NA |
| Gestational age at blood sampling (weeks) | > 0.05 | > 0.05 | > 0.05 | NA |
| BMI (kg/m**^2^)** | > 0.05 | < 0.05 | < 0.0001 | 0.0001 |
| Systolic Blood Pressure (mmHg) | 0.001 | < 0.05 | < 0.0001 | > 0.05 |
| Diastolic Blood Pressure (mmHg) | 0.009 | < 0.05 | > 0.05 | > 0.05 |
| Mean Blood Pressure (mmHg) | 0.001 | < 0.05 | < 0.05 | > 0.05 |
| Fasting glucose (mg/dL) | > 0.05 | > 0.05 | > 0.05 | > 0.05 |
| Triglycerides (mg/dL) | > 0.05 | > 0.05 | > 0.05 | > 0.05 |
| Total cholesterol (mg/dL) | > 0.05 | > 0.05 | > 0.05 | > 0.05 |
| HDL-cholesterol (mg/dL) | > 0.05 | > 0.05 | > 0.05 | > 0.05 |
| LDL-cholesterol (mg/dL) | > 0.05 | > 0.05 | > 0.05 | < 0.05 |
| C Reactive Protein (mg/L) | > 0.05 | < 0.05 | > 0.05 | > 0.05 |
| Fasting insulin (µUI/mL) | > 0.05 | < 0.0001 | > 0.05 | < 0.05 |
| HOMA-IR | > 0.05 | < 0.0001 | > 0.05 | < 0.05 |
| QUICKI | < 0.05 | < 0.0001 | > 0.05 | < 0.0001 |
| TyG index | > 0.05 | > 0.05 | > 0.05 | > 0.05 |
| TG/HDL-c index | > 0.05 | > 0.05 | > 0.05 | < 0.05 |

Non-parametric comparisons. EP = early pregnancy. MP = middle pregnancy. LP = late pregnancy. PP = three months postpartum. EPPE= early pregnancypreeclamptic. MPPE = middle pregnancypreeclamptic. LP = late pregnancy preeclamptic. PP = three months postpartum preeclamptic.BMI (body mass index); HOMA-IR (homeostasis model assessment-estimated insulin resistance); QUICKI (quantitative insulin sensitivity check index); TyG index (triglycerides/glucose); TG/HDL-c index (triglycerides/high-density lipoprotein cholesterol).

**Supplementary table 4. Post-hoc analyses between healthy pregnant women and healthy non-pregnant women**

| **Variables** | **NP vs EP** | **NP vs MP** | **NP vs LP** | **NP vs PP** |
| --- | --- | --- | --- | --- |
| BMI (kg/m**^2^)** | < 0.05 | < 0.0001 | < 0.0001 | < 0.0001 |
| Systolic Blood Pressure (mmHg) | < 0.0001 | < 0.0001 | < 0.0001 | < 0.0001 |
| Diastolic Blood Pressure (mmHg) | < 0.0001 | < 0.0001 | < 0.0001 | < 0.05 |
| Mean Blood Pressure (mmHg) | < 0.0001 | < 0.0001 | < 0.0001 | < 0.05 |
| Fasting glucose (mg/dL) | < 0.0001 | < 0.0001 | < 0.0001 | < 0.05 |
| Triglycerides (mg/dL) | < 0.0001 | < 0.0001 | < 0.0001 | > 0.05 |
| Total cholesterol (mg/dL) | > 0.05 | < 0.0001 | < 0.0001 | < 0.05 |
| HDL-cholesterol (mg/dL) | < 0.05 | < 0.0001 | < 0.0001 | > 0.05 |
| LDL-cholesterol (mg/dL) | > 0.05 | < 0.0001 | < 0.0001 | > 0.05 |
| C Reactive Protein (mg/L) | < 0.0001 | < 0.0001 | < 0.0001 | < 0.0001 |
| Fasting insulin (µUI/mL) | > 0.05 | > 0.05 | < 0.0001 | > 0.05 |
| HOMA-IR | < 0.05 | > 0.05 | > 0.05 | > 0.05 |
| QUICKI | > 0.05 | > 0.05 | > 0.05 | > 0.05 |
| TyG index | < 0.0001 | < 0.0001 | < 0.0001 | > 0.05 |
| TG/HDL-c index | < 0.0001 | < 0.0001 | < 0.0001 | > 0.05 |

Non-parametric comparisons. NP = healthy non-pregnant. EP = early pregnancy. MP = middle pregnancy. LP = late pregnancy. PP = three months postpartum. BMI (body mass index); HOMA-IR (homeostasis model assessment-estimated insulin resistance); QUICKI (quantitative insulin sensitivity check index); TyG index (triglycerides/glucose); TG/HDL-c index (triglycerides/high-density lipoprotein cholesterol).

**Supplementary table 5. Multiple linear regression analysis**

|  | **Dependent variables** | | | | | |
| --- | --- | --- | --- | --- | --- | --- |
|  | **TyG** | | **TG/HDLc** | | **HOMA IR** | |
| Decision coefficient R^2^ | 60.06% | | 33.09% | | 20.75% | |
| p-value (global model) | 0.0000 | | 0.0000 | | 0.0001 | |
| **Independent variables** | **Standard β** | **p-value** | **Standard β** | **p-value** | **Standard β** | **p-value** |
| Intercept (β_0_) | 7.8491 | 0.0000 | 0.955392 | 0.0000 | -1.27172 | 0.0001 |
| HOMA -IR | 0.3571 | 0.0000 | 0.117939 | 0.0242 | - | - |
| Gestational age | 0.0182 | 0.0000 | 0.0696623 | 0.0000 | - | - |
| HDL-cholesterol | -0.0049 | 0.0005 | - | - | - | - |
| LDL-cholesterol | -0.0029 | 0.0000 | - | - | - | - |
| Total Cholesterol | 0.0061 | 0.0000 | - | - | - | - |
| Fasting Insulin | -0.0553 | 0.0001 | - | - | - | - |
| Median BP | -0.0041 | 0.0413 | - | - | - | - |
| BMI | - | - | - | - | 0.1242 | 0.0000 |
| Triglyceride | - | - | - | - | 0.0013 | 0.0214 |

Fitted models by multiple regression after the backward elimination (only the significant effects are left in the model)
